# Adverse event reporting in adult intensive care units and the impact of a multifaceted intervention on drug-related adverse events

**DOI:** 10.1186/2110-5820-2-47

**Published:** 2012-11-22

**Authors:** Alberto Pagnamenta, Giovanni Rabito, Alessandra Arosio, Andreas Perren, Roberto Malacrida, Fabrizio Barazzoni, Guido Domenighetti

**Affiliations:** 1 Department of Intensive Care Medicine of the Ente Ospedaliero Cantonale (EOC), Intensive Care Units of the Regional Hospitals Mendrisio, Locarno, Bellinzona and Lugano, Switzerland; 2 Division of Quality Improvement and Risk Management, Regional Hospital Locarno, Bellinzona, Switzerland; 3 Medical Area Unit of the EOC, Bellinzona, Switzerland

**Keywords:** Adverse events, Medical errors, Patient safety, Quality improvement, Intensive care, Reliability

## Abstract

**Background:**

Adverse events (AEs) frequently occur in intensive care units (ICUs) and affect negatively patient outcomes. Targeted improvement strategies for patient safety are difficult to evaluate because of the intrinsic limitations of reporting crude AE rates. Single interventions influence positively the quality of care, but a multifaceted approach has been tested only in selected cases. The present study was designed to evaluate the rate, types, and contributing factors of emerging AEs and test the hypothesis that a multifaceted intervention on medication might reduce drug-related AEs.

**Methods:**

This is a prospective, multicenter, before-and-after study of adult patients admitted to four ICUs during a 24-month period. Voluntary, anonymous, self-reporting of AEs was performed using a detailed, locally designed questionnaire. The temporal impact of a multifaceted implementation strategy to reduce drug-related AEs was evaluated using the risk-index scores methodology.

**Results:**

A total of 2,047 AEs were reported (32 events per 100 ICU patient admissions and 117.4 events per 1,000 ICU patient days) from 6,404 patients, totaling 17,434 patient days. Nurses submitted the majority of questionnaires (n = 1,781, 87%). AEs were eye-witnessed in 49% (n = 1,003) of cases and occurred preferentially during an elective procedure (n = 1,597, 78%) and on morning shifts (n = 1,003, 49%), with a peak rate occurring around 10 a.m. Drug-related AEs were the most prevalent (n = 984, 48%), mainly as a consequence of incorrect prescriptions. Poor communication among caregivers (n = 776) and noncompliance with internal guidelines (n = 525) were the most prevalent contributing factors for AE occurrence. The majority of AEs (n = 1155, 56.4%) was associated with minimal, temporary harm. Risk-index scores for drug-related AEs decreased from 10.01 ± 2.7 to 8.72 ± 3.52 (absolute risk difference 1.29; 95% confidence interval, 0.88-1.7; *p* < 0.01) following the introduction of the intervention.

**Conclusions:**

AEs occurred in the ICU with a typical diurnal frequency distribution. Medication-related AEs were the most prevalent. By applying the risk-index scores methodology, we were able to demonstrate that our multifaceted implementation strategy focused on medication-related adverse events allowed to decrease drug related incidents.

## Background

At the beginning of this century, the Institute of Medicine published landmark reports on the poor reliability of health care organizations, estimating hospital-based medical errors to be the eighth leading cause of death in the United States [1,2]. Further publications from other countries confirmed that deficiencies in quality of care and in patient safety were not confined to the United States [3-5]. As a consequence, several health care institutions implemented efforts to improve safety at various levels [6,7].

A highly monitored environment and a higher manpower availability in the intensive care unit (ICU) compared with the hospital ward would theoretically imply that the ICU should be the safest and most reliable place within the hospital. The ICU, however, represents a high-risk area for adverse events (AEs) that could occur due to the complexity of care, the large number of interventions performed, and the patients’ fragile medical conditions [8-11]. The occurrence of AEs affects survival rates significantly and independently [12,13]. The high level of monitoring and documentation within the ICU should increase the probability to detect and report AEs. Recognizing AEs, accepting their occurrence, and disclosing them—the later being a crucial step to improve patient safety—are the most important steps to prevent AEs [14].

Presently, no reference method exists to identify AEs [15-17]. Despite several limitations, the system of voluntary reporting of AEs during hospital stay remains a valuable tool to help ICUs to identify safety hazards and learn from deficits, but it does not allow evaluating the progress of improved patient safety [11,15]. Various factors hinder the quantification of the improvement that should follow the implementation of a strategy according to the evolution of the crude rate of AE reports. The risk-index score methodology is an attractive tool to evaluate performance because it is independent of rate-based measures [18], but it has not yet been introduced into the ICU.

Medication-related AEs have been reported to be among the most prevalent types of AE in the ICU [8-11]. Single interventions, such as electronic prescription or involvement of a pharmacist in the direct care of ICU patients, have been reported to affect positively the AE occurrence; a prospectively evaluated, multifaceted implementation program has so far been evaluated only for two selected medications [19]. This investigation therefore aimed at describing the frequency, characteristics, and contributing factors of AEs that emerged during a 24-month period in the ICUs of a large non-University, public, multicenter, teaching hospital-network. We exploited a self-reporting strategy during the hospital stay and tested the hypothesis that a multifaceted intervention focused on medication errors decreased the risk-index scores for drug-related AEs.

## Methods

### Study design

**Figure 1:**
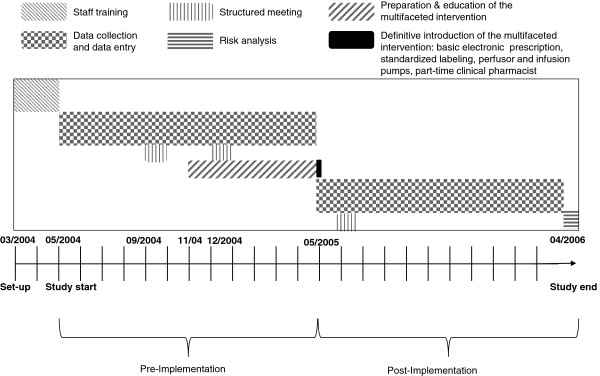
**Study design.** Staff training in adverse events (AE) reporting during 2 months before the pre-implementation period (May 1, 2004 to April 30, 2005). The multifaceted intervention on drug-related AEs was arranged 6 months before its definitive introduction into clinical practice (May 1, 2005). The three structured meetings with the caring staff took place during the same week in the four ICUs.

This was a before-and-after study design. The study first evaluated prospectively the incidence, nature, and contributing factors of emerging AEs in the ICU adopting a detailed, locally developed, self-reporting questionnaire. Thereafter, we tested whether a multifaceted strategy (see later for details) targeted on drug-related AEs would decrease the risk-index scores for drug-related AEs (Figure 1).

Three structured meetings with the care staff during the study period evaluated a specific strategy to increase the reporting rate. This strategy consisted of focused interviews and meetings with the caregivers to address their perspectives on AE reporting in the ICU, identify key barriers to reporting, and propose solutions to increase reporting. These meetings took place during the same week in the four ICUs (Figure 1).

### Study setting

This investigation was performed in four multidisciplinary ICUs [Mendrisio (6 beds), Locarno (7 beds), Bellinzona (8 beds), and Lugano (12 beds); total number of beds: 33] in non-University teaching hospitals in Southern Switzerland. These hospitals with approximately 1,000 beds are integrated in a multisite public hospital as a network (Ente Ospedaliero Cantonale: EOC) providing care to patients in Southern Switzerland and serving a population of 350,000 inhabitants. Because the four institutions have no subunits or intermediate care units, less critically ill patients are admitted to the ICUs, which provide only adult intensive care for medical, surgical, and trauma patients. All ICUs have a closed attending model with hospital-based intensivists who assume the primary responsibility of the patients. At least a junior medical staff is present in each unit and the nursing allocation has a 1:2 ratio. The ICUs are organized with three nurse shifts, rotating at 7:00 a.m., 03:00 p.m., and 11:00 p.m. as well as two or three physician shifts per day.

### Study population and participants

This study included all patients admitted to the ICUs during a 24-month period from May 2004 to April 2006. Patients were followed up until discharge from the ICU or death. Participants included all healthcare workers who were directly involved in or witnessed an AE and reported it. This study was approved by the regional Ethics Committee, which waived the requirement for patient informed consent due to the anonymous nature of all acquired data.

### Data collection

An AE was defined as any unintentional event due to healthcare management, such as human error, organizational failure, or equipment failure that caused or could have caused patient harm. This definition does not necessarily require a prolongation of hospitalization or patient disability nor implies any assumption about the severity of the AE. The AE reporting questionnaire was developed by the study investigators and was first tested in a 2-week pilot phase. Following feedback from the care staff, the clarity and completeness of the questionnaire was improved and the final version was used unaltered during the whole study period (English translation: see Additional file 1). We preferred to develop our own questionnaire to address better our local medical practices. Before the study start, all caregivers in each ICU attended an extensive educational program that provided incentives for AEs reporting to minimize measurement bias. This study was coordinated locally by the chief nurse and the intensivist in charge of each ICU. A study nurse coordinated this trial to help the caregivers maintain high levels of motivation, to provide regular feedback to the staff, as well as to enter the data of the paper-based AE questionnaires into an electronic database for subsequent data analysis.

All AEs were collected continuously. AE reporting was spontaneous, nonpunitive, and confidential as patient and reporter data remained anonymous. Data fields on the questionnaire included professional position and role of reporter, patient characteristics (leading diagnosis, simplified acute physiology score (SAPS II), and the nine equivalents of nursing manpower use score (NEMS) [20]), AE circumstances (when and where the AE occurred), selected AE categories (medication, indwelling lines, communication, airway, equipment, other), and contributing factors (human, team-related, or system-related; multiple selections possible per report). Drug-related AEs were further characterized as incorrect prescription, inconsistency between prescription and administration, wrong time dosage, incorrect administration technique, mistaken time of administration, wrong initial dosage, wrong preparation, or other. Each patient’s basic demographic characteristics were routinely recorded in patient’s medical chart but omitted in the AE questionnaire to maximize anonymity and confidentiality. AEs were classified on a nine-point Likert scale based on their potential to harm (1: no harm; 2: no detectable harm; 3: minimal temporary harm; 4: minimal permanent harm; 5: moderate temporary harm; 6: moderate permanent harm; 7: severe temporary harm; 8: severe permanent harm; 9: death). The likelihood of recurrence of AEs was classified on a five-point Likert scale (1: rare; 2: unlikely; 3: possible; 4: likely; 5: almost certain). The chief nurse and the attending physician reviewed locally each AE questionnaire at weekly intervals to verify data completeness and consistency, to minimize duplicate reporting and to achieve a consensus assessment, reviewing the patient’s medical chart if necessary*.* In case of disagreement, the study-nurse was involved in the adjudication process.

### Multifaceted implementation strategy for drug-related AEs

Considering the high prevalence of medication errors during the first 12 months of this trial as well as reported in other studies [8-11,19], the following medication-related strategies were introduced for the second 12 months: 1) basic electronic prescription without clinical decision support tools; 2) standardized labeling of continuously infused medications; 3) identical models of perfusors and infusion pumps; 4) partial involvement of a pharmacist in the direct care of ICU patients (part-time pharmacist who checked all electronic prescriptions for drug-dosing adjustments for hepatic and/or renal dysfunction, drug interactions, and compatibility checking of the patient’s intravenous medications). These four measures were easy to implement in first line as drug-related AEs represent a multistep problem and require different approaches. The optimal preparation of the caring staff required only a 6-month run-in period for the measures to be introduced before definitely becoming part of the clinical practice (Figure 1). Before the new electronic prescribing process, drug prescription was handwritten, in most cases by a trainee after the clinical daily round. The electronic prescription took place during the multidisciplinary clinical round, thus allowing direct check of prescriptions, better communication between the different caregivers and integration of treatment plans.

### Data analysis

Paper-based data were first entered into a Microsoft Excel database and then exported to the SPSS® package (SPSS Version 18, Chicago, IL). Reporting rates were calculated as number of events reported per 100 ICU-patient admissions and 1,000 ICU-patient days. The distribution of daily AEs was evaluated using an analysis of variance. For risk assessment, a risk matrix was adopted in accordance with the guidelines of the National Patient Safety Agency of the UK [18]. A risk-index score was obtained by multiplying the potential harm of a given AE by its likelihood of recurrence and graded as follows: 1–3: low risk; 4–6: moderate risk; 7–15: high risk; 16–45: extreme risk. Risk-index scores are presented as mean ± standard deviation (SD) after testing for normality of the sample distribution using the Kolmogorov-Smirnov test. The risk-index score analysis was performed on the most prevalent AE categories (medication, indwelling lines, communication/planning, and airway-related). The mean risk-index values of the first and the subsequent 12 months (before and after the introduction of the multifaceted intervention) were compared using the Student’s *t* test, and the absolute risk differences are presented with the corresponding 95% confidence intervals (CIs). All tests were two-sided, and *p* values <0.05 were considered statistically significant. The crude monthly AE reporting rate was evaluated using a general linear regression model with repeated measures.

## Results

During the study period, 6,404 adult patients were admitted to our ICUs, representing 17,434 patient days (Table 1).

**Table 1:** Patient characteristics

**Variable**	**First 12 months**	**Last 12 months**	**Whole study period**
No. of patients	3111	3293	6404
Age (yr), mean ± SD	64.6 ± 17.5	63.2 ± 18.2	63.8 ± 17.9
Male sex, n (%)	1743 (56)	1975 (60)	3718 (58)
SAPS II at admission, mean ± SD	34.2 ± 17.3	36.8 ± 17.8	35.5 ± 17.6
Type of admission, n (%)			
Medical	2180 (70)	2173 (66)	4353 (68)
Scheduled surgery	465 (15)	590 (18)	1055 (16.5)
Unscheduled surgery	466 (15)	530 (17)	996 (15.5)
Principal reason for admission to unit, n (%)			
Cardiovascular disorders	872 (28)	985 (30)	1857 (29)
Respiratory disorders	531 (17)	461 (14)	992 (15.5)
Neurological disorders	373 (12)	421 (12.8)	794 (12.4)
Gastrointestinal disorders	292 (9.4)	336 (10.2)	628 (9.8)
Trauma	311 (10)	290 (8.8)	601 (9.4)
Intoxication	155 (5)	165 (5)	320 (5)
Metabolic disorders	124 (4)	132 (4)	256 (4)
Other	452 (14.5)	504 (15.3)	956 (14.9)
Length of ICU stay, days			
Mean ± SD	2.8 ± 5.3	2.9 ± 5.2	2.8 ± 5.3
Median (25th percentile, 75th percentile)	1.4 (1.0, 3)	1.1(0.8, 2.5)	1.2 (0.8, 2.7)
ICU mortality, n (%)	182 (5.8)	176 (5.3)	358 (5.6)

Data are presented for the pre-implementation period (first 12 months) for the post-implementation period (last 12 months), and for the whole study period (24 months). ICU, intensive care unit; SAPS, simplified acute physiology score; SD, standard deviation.

### AE reporting frequency and characteristics

**Figure 2:**
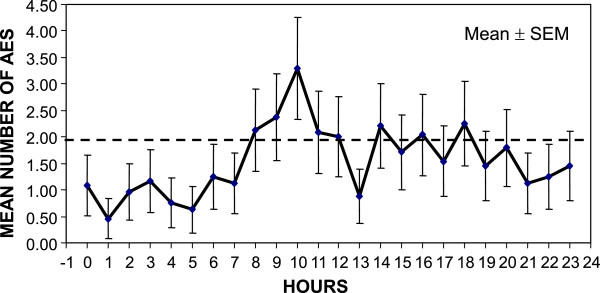
**Distribution of adverse events (AEs) during the day.** Most reported AEs occurred between 08:00 and 12:00 a.m., with a peak around 10:00 am. Horizontal dashed line (at 1.8145 AEs/hour) indicates the limit of statistical significant among comparisons of means AEs per hour. SEM, standard error of the mean.

A total of 2,047 AEs were recorded corresponding to 32 events per 100 ICU-patient admissions and for 117.4 events per 1,000 ICU-patient days (Table 2). The SAPS II score was recorded in 27% of AE (n = 553) questionnaires. Most AEs occurred during elective procedures (n = 1,597, 78%) and the majority of AEs took place within the ICU (n = 1,862, 91%). AE reporting was equally distributed between working days and weekends (mean AE ± SD: working days 2.84 ± 1.11 and weekends 2.69 ± 1.28; *p* = 0.51); 49.3% (n = 1,009) of the reported AEs occurred during the morning shift, 30.3% (n = 620) during the evening shift, and 20.4% (n = 418) during the night shift. Most AEs occurred between 08:00 and 12:00 a.m. with a peak around 10:00 am (Figure 2). The most frequent drug-related AEs were associated with incorrect prescriptions (Table 3).

**Table 2:** Adverse events characteristics

**Variable**	**First 12 months**	**Last 12 months**	**Whole study period**
No. of AEs	1071	976	2047
Person reporting, n (%)			
Nurse	869 (81)	888 (91)	1757 (86)
MD	190 (18)	79 (8)	269 (13)
Other	12 (1)	9 (1)	21 (1)
Role of reporter, n (%)			
Eye-witnessed the AE	494 (46)	509 (52)	1003 (49)
Involved in AE occurrence	395 (37)	321 (33)	716 (35)
Was called for help	138 (13)	129 (13)	267 (13)
Missing data	30 (3)	31 (3)	61 (3)
Patients NEMS in AEs questionnaire, n (%)			
NEMS ≥25	227 (21)	203 (21)	430 (21)
NEMS 22-24	450 (42)	430 (44)	880 (43)
NEMS 14-21	302 (28)	251 (26)	553 (27)
NEMS <14	42(4)	40 (4)	82 (4)
NEMS, missing data	62 (6)	40 (4)	102 (5)
Patients leading diagnosis in AEs questionnaire, n (%)			
>1 leading diagnosis	446 (42)	332 (34)	778 (38)
Respiratory disorders	204 (19)	213 (22)	409 (20)
Cardiovascular disorders	176 (16)	172 (18)	348 (17)
Neurological disorders	77 (7)	66 (7)	143 (7)
Postoperative	60 (6)	63 (6)	123 (6)
Other	134 (13)	112 (11)	246 (12)
AE categories, n (%)			
Drug-related	506 (47)	478 (49)	984 (48)
Indwelling lines, catheters and drains	135 (13)	110 (11)	245 (12)
Communication and planning	107 (10)	104 (11)	213 (10)
Airway	100 (9)	88 (9)	188 (9)
Equipment	75 (7)	89 (9)	164 (8)
Other procedures (bronchoscopy, EGD, electrical cardioversion…)	75 (7)	60 (6)	135 (7)
Others (documentation, positioning…)	64 (6)	54 (6)	118 (6)

Data are presented for the pre-implementation period (first 12 months), for the post-implementation period (last 12 months), and for the whole study period (24 months). AE, adverse event; EGD, esophagogastroduodenoscopy; NEMS, nine equivalents of nursing manpower use score; MD, medical doctor.

**Table 3:** Characteristics of drug-related adverse events

**Variable**	**Data**
No. of drug-related AEs	984
Incorrect prescription, n (%)	226 (23)
Inconsistency between prescription and administration, n (%)	207 (21)
Wrong time dosage, n (%)	197 (20)
Incorrect administration technique, n (%)	128 (13)
Mistaken time of administration, n (%)	98 (10)
Wrong initial dosage, n (%)	59 (6)
Wrong preparation, n (%)	49 (5)
Other, n (%)	20 (2)

AE, adverse event.

### Contributing factors of reported AEs

The contributing factors associated with AEs were further classified as human, team, or system factors. Among human factors, noncompliance with internal guidelines, policies, or checklists (n = 525) were the most prevalent categories, followed by wrong procedure planning (n = 481), attention deficit without sleep deprivation (n = 464), and elevated workload (n = 433). Among team-related factors communication failure between nurses and physicians was evident in 420 cases, communication failure within the nursing staff in 356 cases. Time pressure due to organizational problems and insufficient staffing were the most prevalent categories (217 and 183 reports, respectively) of the system-related factors.

### Description of harm

On the nine-point scale of potential harm, 1,155 AEs (56.4%) were classified as level 3 (minimal temporary harm), 302 (14.8%) as level 4 (minimal permanent harm), and 265 (12.9%) as level 2 (no detectable harm). No AEs leading to the patient death were recorded, but five AEs (0.2%) were related to severe permanent harm (level 8).

### Effects of the multifaceted intervention

The mean risk-index score for medication errors improved from the first 12-month period to the second 12-month period from 10.01 ± 2.7 to 8.72 ± 3.52 (absolute risk difference 1.29; 95% CI 0.88-1.7; *p* < 0.01); the mean risk-index score for communication improved as well. Contrarily, the mean risk-index scores for airway-related and for indwelling lines-related AEs did not change over time (Table 4).

**Table 4:** Risk-index score analysis as a function of time: before (first 12-month period) and after (last 12-month period) the introduction of a multifaceted strategy targeting medication

**Risk index scores**	**First 12 months**	**Last 12 months**	**Absolute risk difference (95% CI)**	***p *value**
Medication, mean ± SD	10.01 ± 2.7	8.72 ± 3.52	1.29 (0.88-1.7)	<0.01
Airway, mean ± SD	9.22 ± 1.52	8.69 ± 1.83	0.53 (−0.92-1.98)	0.46
Indwelling lines, mean ± SD	10.41 ± 2.79	9.2 ± 3.82	1.21 (−0.41-2.47)	0.058
Communication, mean ± SD	9.29 ± 2.78	7.03 ± 3.22	2.26 (1.1-3.42)	<0.01

SD, standard deviation; CI, confidence interval.

### Effects of the structured meetings

**Figure 3:**
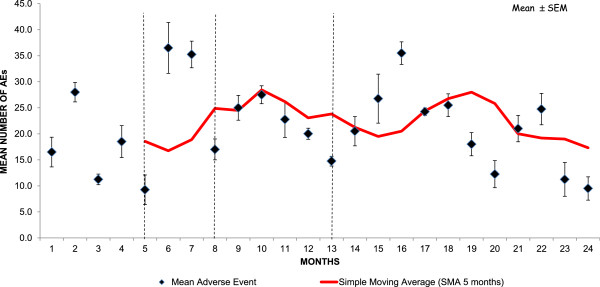
**Mean monthly reporting rates of adverse events (AEs) during the entire study period (24 months).** Vertical dashed lines indicate the occurrence of structured meetings with the care staff. After each meeting, an increase in AE reporting occurred.

AE reporting increased in the months following each structured meeting with the care staff (Figure 3).

## Discussion

Concurring with many previous reports [8-11,21,22], the present study confirmed that AEs are frequently experienced by ICU patients. Our study showed clearly that risk-index scores for drug-related AEs decreased as a consequence of the multifaceted intervention strategy on medication that we applied. Despite the intrinsic limitations of self-reporting methodologies for evaluating performance, we think that a risk-index score analysis represents a useful tool to evaluate targeted improvement strategies.

AE incidence rates are difficult to compare among studies, in part because of different AE reporting strategies. Despite implementation of a single methodology, the Sentinel Events Evaluation (SEE) 1 study reported medication errors by unit staff at a rate of 10.5 per 100 patient days [23], whereas a value of 74.5 per 100 patient days was documented in the SEE 2 study [24]. This difference is probably related to the fact that the SEE 1 study captured errors in five selected AE categories, whereas the SEE 2 study focused on medication errors only.

As documented in other similar studies, our reporting rates differed among healthcare worker groups [10,11]. ICU nurses reported most of the AEs reflecting the different numbers and types of activities performed by distinct caregiver groups. Contrary to popular belief, most of the AEs in our study occurred during elective procedures, as in the multicenter SEE 2 study [24]. Elective procedures may be considerably improved by adopting educational interventions and protocol implementation. Single-center and multicenter studies have exhibited similar diurnal distributions of AEs, with a peak in morning caregiver activities [8,17]. This observation highlights the need to recognize these variations throughout the day and to identify the corresponding opportunities for error, which will promote better planning for and allocation of resources to decrease risk.

The characteristics of the AEs in our study are consistent with previous reports that suggest a common pattern of AEs in the ICU [8-11,23]. Drug-related AEs are the most common; these errors are frequently detected and reported because medication administration is a multistep procedure that requires correct prescription and administration of the right drug to the right patient at the right dose via the right route at the right time [21,22,24]. Our most frequent medication-based AEs were related to incorrect prescriptions. The practice of handwritten and sometimes only verbally communicated medical prescription in our ICUs may partly explain this finding. Furthermore, the prescription was annotated by a trainee in most of the AE cases. This calls for a better supervision when prescriptions are ordered verbally. As previously reported [21,22,24], many of these medication-related AEs can potentially be prevented using adequate information and communication technologies, which in turn, however, may introduce new hazards requiring further investigation [25]. In a stepwise multivariate logistic regression analysis, Valentin et al. found that physician electronic prescribing was associated with reduced odds for the occurrence of a medication-related AE [24]. A basic computerized prescribing tool can be implemented in combination with clinical decision support for correct drug dosing, a drug/laboratory value check, and a drug/drug interaction check. Finally, the involvement of a pharmacist in the direct care of ICU patients has been shown not only to positively impact patient outcome, but also to reduce costs [26,27]. Noncompliance with internal guidelines, policies, and checklists was frequently reported in our study as a personal-related contributing factor for AEs, underlying the urgent need to improve continuous education of our ICU personnel. Poor communication among the care staff has been repeatedly identified as a contributing factor to many AEs [28,29] and also has been observed in our study. Possible solutions may be achieved by improving interdisciplinary communication during bedside rounds in the ICUs, which was previously associated with reduced AE rates [30]. Furthermore, communication skills training is needed to create a favorable atmosphere of trust and respect allowing open interaction as well as creating a positive unit climate, which has recently been shown to maximize patient safety [31].

Our multifaceted, medication-focused intervention was associated with reduced risk-index scores for drug-related AEs, although we cannot ultimately confirm any causal relationship despite the consistency, plausibility, and temporality. Moreover, the unchanged risk-index scores for airway-related AEs and indwelling lines-related AEs supports the hypothesis that our multifaceted intervention plan positively affected medication-related AE risk-index scores. Because we did not introduce any specific strategy for mitigating communication-related AEs, we assume that the new electronic prescribing process affected indirectly the observed improved risk-index score in this category. Computerized physician order entry performed during the multidisciplinary clinical round allowed a better communication and integration of treatment plans between caregivers. In a cluster-randomized, crossover study, Garrouste-Ogeas et al. demonstrated that multifaceted safety programs decreased insulin administration errors [19]. Contrary to our study, their trial focused on two selected medications (insulin and anticoagulant) at two selected stages (prescription and administration). Furthermore, AE reporting resulted from a combination of an external observer with medical chart review and significant Hawthorne effects (bias following a change in behavior under observation) were observed by the authors. Compared with our study, the cluster-randomized, crossover design used by Garrouste-Ogeas et al. has obviously several strengths (no bias arising from regression to the mean and no historical bias).

We also have demonstrated in agreement with a previous report [11] that AE reporting increased after structured meetings with the care staff that addressed the staff’s perspectives on AE reporting. Our improved post-meeting reporting rate decreased in the following months, suggesting that regular meetings at closer intervals are needed to maintain a high and constant reporting rate.

This trial has several limitations. First, basic patient demographic characteristics were not mentioned in the AE report to maximize anonymity and confidentiality. As a consequence, we did not document the number of patients suffering an AE, but only the total number of events assigned to different categories. This limitation precludes the calculation of odds ratios and a more sophisticated statistical analysis. Second, the survey was performed in four multidisciplinary ICUs of non-University teaching hospitals using a locally developed AE questionnaire. Therefore, our results may not be generalizable to other ICUs with different characteristics (patient types, severity of illness, staffing pattern). Third, the nurses reported most of the AEs, inducing a possible reporting bias. However, this observation is consistent with previous reports [10,11], reflecting the larger time specific caregivers spend in direct patient care. A before-and-after study design such as ours that evaluates a multifaceted implementation strategy has the potential risk of historical bias and other possible external confounders [32]. These limitations could be avoided with a randomized study design, but this was impractical in our ICUs. Finally, our study focused on AE reporting rather than on patient safety. In this context, it is important to remember that AE reporting is the first step in safety-improvement strategies [15]; the SEE 2 study revealed that an existing critical incident reporting system was an independent predictor for a decreased risk of parenteral medication-based AEs at the administration stage [24].

The strengths of our study include the multicenter design, the long study period, the prospective self-reporting by caregivers during hospital stay, the utilization of a detailed questionnaire allowing better characterization of AE occurrence, and the use of the risk-index score methodology. This methodology may enable monitoring of the results of a specific implementation plan. Reporting of the crude AE rates before and after an implementation cannot be used to evaluate progress in patient safety for several reasons, including nonrandom sample reporting from an unknown probability distribution, a reporting bias of unknown magnitude and direction as well as an unknown at-risk population [14]. A risk-index score as a function of time may represent a useful tool for the assessment of targeted improvement interventions, given its use of a non-rate-based measure. Despite defining consequences as objectively as possible, it is inevitable that scoring the consequences of some risks will involve a degree of subjectivity [18]. It is important that effective, practical training and relevant examples form a part of the implementation of any risk-assessment system to maximize scoring consistency across the organization.

As in other investigations of AEs in the ICU, we had no “gold-standard” method to detect AEs. The use of external observers [8,19,21] may be considered a reference method for capturing AEs, but this strategy appears to consume a large number of resources and suffers from the Hawthorne effect. The AE self-reporting method provides a detailed description of the AE and identifies a large number of preventable incidents, but it has the risk of selection bias, underreporting and some degree of Hawthorne effect, whereas retrospective medical chart review provides less contextual information for an AE and identifies fewer preventable incidents. A combination of different data-gathering methods is likely to be optimal. However, it is usually accepted that it is preferable to reduce the quantity but not the quality of data collection [32].

## Conclusions

This study once again demonstrates that adverse events are common in ICU and drug-related AE is the most prevalent. Errors have a typical diurnal frequency distribution calling for a corresponding human resources allocation. We identified several potential prevention strategies that could be relatively easily introduced in clinical practice to improve patient safety. By applying the risk-index scores methodology, we were able to demonstrate that our multifaceted implementation strategy focused on medication-related adverse events, allowed to decrease drug-related incidents.

## Competing interests

The authors declare that they have no competing interests.

## Authors’ contribution

GD conceived the study. AP, AA, APe, RM, FB, and GD participated in the design of the study. All authors participated in the acquisition of data. AP, AA, GR, and GD performed the statistical analysis. AP, GR, APe, and GD drafted the manuscript. All authors revised the manuscript for important intellectual content and have given final approval for publication of this version. All authors read and approved the final manuscript.

## Supplementary Material

Additional file 1 Intensive care adverse events (AE) monitoring: English translation of the original AE reporting questionnaire. Click here for file
