# Supplementary material for: Adverse event reporting in adult intensive care units and the impact of a multifaceted intervention on drug-related adverse events
Source: Ann Intensive Care. 2012 Nov 22;2:47. doi: 10.1186/2110-5820-2-47 (PMC3526522; doi:10.1186/2110-5820-2-47)
Supplement: Additional file 1 — Intensive care adverse events (AE) monitoring: English translation of the original AE reporting questionnaire. [file 2110-5820-2-47-S1.pdf]

Intensive Care Adverse Events (AE) Monitoring

Q01 - Who reported the AE?

Q01a - Professional position

1 ☐ Medical doctor
2 ☐ Nurse
3 ☐ Other

Q01b - Role of reporter

1 ☐ who reports was involved in the AE occurrence
2 ☐ who reports was called for help
3 ☐ who reports eye-witnessed the AE

Q02 - Patient's characteristics

Q02a - Nursing patient workload

1 ☐ 1a
2 ☐ 1b
3 ☐ 2
4 ☐ 3
5 ☐ Don't know

Q02b - Leading diagnosis

1 ☐ respiratory disorders
2 ☐ cardiovascular disorders
3 ☐ trauma
4 ☐ neurological disorders
5 ☐ postoperative
8 ☐ gastrointestinal disorders
9 ☐ urogenital disorders
10 ☐ blood disorders

Q02c - SAPS II score

1 ☐ 0-30
2 ☐ 31-40
3 ☐ 41- 50
4 ☐ 51-60
5 ☐ 61-70
6 ☐ 71-80
7 ☐ 81-120
8 ☐ Don't know

Q03 - Details of adverse event (AE)

Q03a - Procedures

1 ☐ elective procedures
2 ☐ emergency procedures

Q03b - Where the AE occurred

1 ☐ Intensive care unit (ICU)
2 ☐ outside the ICU

Q03c - When the AE occurred

1 ☐ weekend or holiday
2 ☐ working days

Q03d - Timing of the AE (between 00 and 23)

☐☐ (hours)

Q03f - Diagnostic and therapeutic invasive procedures

1 ☐ airway
2 ☐ indwelling lines, catheters and drains
3 ☐ other procedures (bronchoscopy, EGD, electrical cardioversion,…)

Q03g - Diagnostic and therapeutic non-invasive procedures

1 ☐ medication
- 1.1 ☐ incorrect prescription
- 1.2 ☐ wrong preparation
- 1.3 ☐ wrong initial dosage
- 1.4 ☐ incorrect administration technique
- 1.5 ☐ inconsistency between prescription and administration
- 1.6 ☐ mistaken time of administration
- 1.7 ☐ wrong time dosage
- 1.8 ☐ other

2 ☐ communication and planning
3 ☐ other (positioning, physiotherapy, documentation, radiology examens,…)
4 ☐ equipment

Q04 – Who (agent) caused the AE?

Q04a - Workload of the agent who caused the AE

hours worked until the AE occurred ☐☐ (hours)

1 ☐ Don't know

Q04b - Professional position

1 ☐ Medical doctor
2 ☐ Nurse
3 ☐ Other --

Q04c – Educational degree

1 ☐ In training
2 ☐ Certified
3 ☐ Other --
4 ☐ Don't know

Q05 – Brief description of the AE

Description of the situation before the AE occurred, without the details which may identify the agent, the reporter or the patient.

____

____

____

____

____

____

Q06 – Brief description of response to the AE

Description of management of the case (of the situation) after the AE and corrections proposed.

____

____

____

____

____

____

____

____

Q07 – Consequences of the AE (result)
(if already known; multiple answers are possible)

1 ☐ Result independent of the AE
2 ☐ Patient dissatisfaction
3 ☐ Interruption of treatment
4 ☐ Extension of stay in Intensive Care
5 ☐ Extension of hospital stay
6 ☐ Transfer (within or outside the hospital)
7 ☐ Low level of morbidity
8 ☐ High level of morbidity
9 ☐ Disability
10 ☐ Death
11 ☐ Don't know

Q08 – Avoidability of the AE

Q 08a – The AE was

1 ☐ Avoidable
2 ☐ Unavoidable
3 ☐ Don't know

Q09 – Assessment of the AE
(multiple answers are possible)

a – What caused the AE?

Q 09a1 – Human factors

1 ☐ Reduction in attention without a sleep deficit
2 ☐ Reduction in attention with a sleep deficit
3 ☐ Illness
4 ☐ Insufficient professional knowledge/practical ability
5 ☐ Very high workload
6 ☐ Very busy private life
7 ☐ Incorrect planning of the procedure
8 ☐ Failure to follow guidelines, directives or checklists
9 ☐ Don't know

Q09a2 –Team-related factors: insufficient communication

1 ☐ Between members of the nursing team
2 ☐ Between members of the nursing team and the patient
3 ☐ Between members of the medical team
4 ☐ Between members of the medical team and the patient
5 ☐ Between members of the nursing team and family members
6 ☐ Between members of the medical team and family members
7 ☐ Between members of the medical and nursing teams
8 ☐ Between members of the healthcare team (nursing/medical staff) and who sent the patient
9 ☐ Don't know

Q09a3 – System factors

1 ☐ Not enough staff
2 ☐ Not the usual environment
3 ☐ Time pressure due to a situation dangerous to the patient
4 ☐ Time pressure due to organisational problems
5 ☐ Technical/equipment problems
6 ☐ Managerial/hierarchical problems
7 ☐ Don't know

b – What factors made it possible to manage the problem (strategies for solving the problem)?

Q09b1 – Human factors

1 ☐ Adequate knowledge
2 ☐ Practical ability
3 ☐ Experience
4 ☐ Attention paid to the situation
5 ☐ Use of suitable algorithms
6 ☐ Don't know

Q09b2 – Team-related factors: good communication (consultation)

1 ☐ Between members of the nursing team
2 ☐ Between members of the nursing team and the patient
3 ☐ Between members of the medical team
4 ☐ Between members of the medical team and the patient
5 ☐ Between members of the nursing team and family members
6 ☐ Between members of the medical team and family members
7 ☐ Between members of the medical and nursing teams
8 ☐ Between members of the healthcare team (nursing/medical staff) and who sent the patient
9 ☐ Don't know

Q09b3 – Systemic factors

1 ☐ Good consultation or habits of working together between members of the healthcare team
2 ☐ Additional monitoring/equipment
3 ☐ Replacement of the monitoring/equipment
4 ☐ Additional staff
5 ☐ Replacement of staff
6 ☐ Don't know
